# The Common yet Different Journey: Role Transition Among Hospital‐Based Advanced Practice Nurses in Mainland China: A Grounded Theory Study

**DOI:** 10.1155/jonm/1897994

**Published:** 2026-09-25

**Authors:** Wei Tan, Qin Hu, Shanshan Liu, Yan Jiang

**Affiliations:** ^1^ Evidence-Based Nursing Center, West China Hospital, Sichuan University/West China School of Nursing, Sichuan University, Chengdu, China, scu.edu.cn; ^2^ Sichuan Provincial Engineering Research Center of Medical Nursing Equipment and Materials, Chengdu, China; ^3^ Department of Nursing, West China Hospital, Sichuan University/West China School of Nursing, Sichuan University, Chengdu, China, scu.edu.cn

**Keywords:** advanced practice nurses, constructivist grounded theory, experience, mainland China, qualitative research, transition

## Abstract

**Aims:**

To explore role transition among hospital‐based advanced practice nurses (APNs) in mainland China and develop a substantive theory explaining how they move into and enact advanced practice roles.

**Background:**

APN roles have been introduced internationally to expand nursing practice and improve healthcare delivery. Transition into these roles is challenging because nurses must develop new competencies, assume expanded responsibilities and reconstruct professional identity. This process may be particularly complex in mainland China, where APN roles are largely institutionally based and clinically oriented, with role structures and practice models continuing to evolve. In this emerging APN context, examining how nurses adapt to and enact advanced practice roles provides important insight into the processes underlying role transition. However, empirical evidence on APN role transition in mainland China remains limited.

**Methods:**

A constructivist grounded theory approach was used. Purposive and theoretical sampling recruited 33 APNs in mainland China. Data were collected through longitudinal and retrospective interviews. Data analysis involved initial, focused and theoretical coding, supported by constant comparison and memo‐writing. Synchronic and diachronic strategies examined patterns and changes over time.

**Results:**

The core category “the common yet different journey” emerged, reflecting common transition stages, different transition trajectories and distinct transition outcomes. Four transition stages were identified: role confusion, role exploration, role integration and continuous development. APNs moved through these stages along five trajectories: sequential, leap, back‐and‐forth, stagnant and divergent, corresponding to role maturity, role stagnation, or role divergence. Individual, organisational and social conditions shaped these trajectories through a recursive process of role appraisal, adaptive negotiation and recognition feedback.

**Conclusions:**

This study offers a contextually grounded explanation of role transition among hospital‐based APNs in mainland China. The findings show that APN transition is a dynamic process shaped by interactions between individual development and contextual conditions.

**Implications for Nursing Management:**

The substantive theory provides a basis for understanding APN role transition. Managers should provide staged and tailored support. Mechanisms to document and evaluate APN contributions are needed to strengthen organisational recognition and inform APN role and policy development.

## 1. Introduction

As global healthcare demands continue to rise and shortages of healthcare professionals remain a major challenge [1], advanced practice nursing roles have been introduced in many countries and regions, and their value has been increasingly recognised [2, 3]. Internationally, the International Council of Nurses defines an advanced practice nurse (APN) as a generalist or specialised nurse who has acquired, through additional graduate education at a minimum of a master’s degree, expert knowledge base, complex decision‐making skills and clinical competencies for advanced nursing practice [4]. Nurse practitioners and clinical nurse specialists are two commonly recognised APN roles. Evidence suggests that APN roles can improve care quality, enhance service delivery and contribute to better patient outcomes [5–7].

However, transition into a new role is often a turbulent and challenging process [8].

For APNs, this transition involves adapting to new professional expectations, establishing role legitimacy and integrating advanced practice into existing healthcare systems [9]. This process also involves changes in professional identity, role expectations and perceived autonomy [10, 11]. Empirical studies have further illustrated these transition difficulties. In Barnes’s survey of 352 nurse practitioners, the mean role transition score was 48.92 out of a possible 77 [12]. Similarly, Ares’s study of 68 clinical nurse specialists reported low career commitment, with 76.4% experiencing imposter phenomenon accompanied by anxiety, frustration and depression [13]. Chen and Lin also found that 51.2% of nurse practitioners experienced personal burnout [14].

To explain and support APN role transition, scholars have developed several transition models and intervention strategies [9, 15]. For example, Brown and Olshansky’s model, “From Limbo to Legitimacy,” described the common transition pathway of primary care nurse practitioners [16]. Educational interventions based on this model have been used to enhance nurses’ confidence and attitudes toward advanced practice roles [17]. However, most of these models were developed in formally regulated APN systems, making it necessary to examine APN role transition in emerging contexts such as mainland China.

In mainland China, advanced practice nursing remains at an early stage of development. Although the International Council of Nurses recommends master’s‐level preparation for APNs, it also recognises that APN characteristics are shaped by the contexts in which APNs are credentialed to practise. Accordingly, APN preparation pathways vary across countries, ranging from continuing education and post‐registration programmes to master’s‐level education in some emerging systems [3]. Reflecting this contextual variation, APN roles in mainland China are currently largely institutionally based and clinically oriented, with preparation provided through both master’s‐level programmes and structured post‐registration training offered or recognised by nursing associations, higher education institutions and large teaching hospitals [18–20]. APN scope of practice and professional authority remain largely institutionally determined.

Although APN roles in mainland China are still developing, nurses entering institutionally designated advanced practice roles nevertheless experience a substantive role transition [8]. In this study, role transition refers to the process through which nurses move into and enact APN roles involving expanded responsibilities, evolving boundaries and new expectations for advanced practice [21]. Examining this transition provides insight into how APN roles are interpreted, enacted and negotiated while role structures and practice expectations continue to develop.

Beyond role and organisational challenges, sociocultural expectations, public perceptions and traditional views of nursing as task‐oriented work may further influence the recognition and development of advanced practice nursing in mainland China [22, 23]. Although increasing numbers of nurses are entering institutionally designated APN roles in mainland China, empirical evidence on how nurses experience and navigate the transition into APN roles remains limited. Therefore, this study aimed to explore role transition among hospital‐based APNs in mainland China and develop a substantive theory explaining how they move into and enact advanced practice roles.

## 2. Methods

### 2.1. Study Design

This study used a constructivist grounded theory approach based on the principles of symbolic interactionism. The longitudinal constructivist grounded theory design was planned prospectively from study inception. This approach emphasises flexibility and is particularly suited to exploring complex social phenomena that are not well explained by existing theories [24]. It enables the co‐construction of meaning through an iterative process, involving both the participants′ personal experiences and the researcher’s interpretive analysis [25]. This report adheres to the Consolidated Criteria for Reporting Qualitative Research (COREQ) [26] (Supporting Information 1).

### 2.2. Setting and Participants

Participants were recruited from large public hospitals in mainland China. Because a national regulatory framework for APN has not yet been established in mainland China, APNs in this study were operationalised for the purposes of participant eligibility. Specifically, APNs were defined as registered nurses who: (1) had completed a structured advanced practice nursing training programme provided or recognised by a nursing association, higher education institution or large teaching hospital; (2) had been designated or assigned by their employing institution to undertake an advanced practice nursing role and (3) were currently undertaking expanded clinical responsibilities beyond the usual scope of general nursing practice. Nurses who had completed relevant training but were not currently practising in an advanced role were not eligible.

Participants’ responsibilities varied according to clinical speciality and institutional role arrangements, but they generally centred on complex patient care, management of critically ill patients, early identification of acute clinical deterioration, nurse‐led clinic consultations, specialist nursing consultations, interprofessional collaboration and care coordination, quality‐improvement activities, clinical teaching and evidence‐based practice or research. These responsibilities were broadly aligned with the nationally developed APN core competency framework in mainland China, which encompasses six domains: clinical care, consultation, coordination, leadership, teaching and research [27]. Participants did not have nationally granted independent authority to make medical diagnoses or prescribe medications; when medication‐related decisions were involved, they contributed in collaboration with physicians.

Purposive sampling was initially used to recruit participants with direct experience of APN role transition and to capture variation in professional backgrounds. As data collection and analysis progressed, emerging categories guided theoretical sampling to further develop category properties, explore variations and clarify relationships among categories.

### 2.3. Data Collection

Previous studies have identified the first year of practice as a critical period during which APNs gradually adapt to a new professional identity [16, 28, 29]. However, given the context‐specific development of advanced practice nursing in China, the duration and pattern of role transition remain uncertain. Novice APNs who had practised for no more than 1 month at baseline were therefore followed through three interviews over 1 year to capture changes in their experiences over time [9, 29]. Following completion and preliminary analysis of the longitudinal interviews, APNs with at least 1 year of practice experience were recruited for a single retrospective interview to provide accounts of their first‐year transition experiences and current advanced practice [30] (Table 1).

**TABLE 1:** Data collection schedule.

Participant group	Interview design	Number of interviews	Interview timing
Novice APNs with ≤ 1 month of practice at baseline	Longitudinal	3	T1: approximately 1 month; T2: approximately 6 months; T3: approximately 12 months of practice

Experienced APNs with ≥ 1 year of practice	Single retrospective interview	1	/

Abbreviation: APN, advanced practice nurse.

Based on a literature review, research‐team discussions and pilot interviews with two participants, semistructured interview guides were developed. The guides were refined through concurrent data collection and analysis. In particular, the second and third longitudinal interview guides focused on changes since the previous interview, critical events, turning points and factors influencing role transition. The retrospective interview guide for experienced APNs focused on their first year of advanced practice and current role enactment, using temporal probes to guide recall of early expectations, major challenges, critical events, turning points, coping strategies and changes in role understanding. The interview guides are provided in Supporting Information 2.

All interviews were conducted by the first author, either face to face or virtually. Face‐to‐face interviews were conducted in a hospital conference room to ensure confidentiality and minimise interruptions. All interviews were audio‐recorded in Chinese, and field notes documented participants’ nonverbal expressions, contextual observations and the interviewer’s reflections.

### 2.4. Data Analysis

Data analysis and data collection were conducted concurrently and iteratively by the first author, following the principles of constructivist grounded theory [24]. Initial coding was conducted line by line to identify actions and meanings related to APNs’ role transition. During focused coding, significant and recurrent initial codes were compared and synthesised into higher‐level categories. Theoretical coding was then used to explore relationships among categories and integrate them into an explanatory theoretical model.

Baseline (T1) interview data from 19 novice APNs were also analysed in a previously published descriptive phenomenological study of early transition experiences [31]; subsequent longitudinal and retrospective interviews were not included in that study. The present grounded‐theory analysis examined changes and variations across participants and time points to develop an explanatory theory of APN role transition.

Memo‐writing was integrated throughout the analysis. For the longitudinal data from novice APNs, synchronic and diachronic analyses were used to examine both cross‐sectional patterns and within‐participant changes over time. In synchronic analysis, round‐specific comparison tables were used to identify common and differing experiences across participants within each interview round. In diachronic analysis, a longitudinal case summary was maintained for each participant and updated after each round to trace key experiences, critical events, changes over time and emerging temporal patterns [32, 33]. Retrospective interviews with experienced APNs were incorporated into the ongoing constant comparison process and compared with the longitudinal data to elaborate and refine category properties, variations and relationships, particularly in relation to later‐stage role development. Through iterative comparison across cases and time points, patterns of role transition were progressively conceptualised during theoretical coding, including variations in movement across transition stages and the development of distinct transition trajectories. Analytic memos documented category development across both data sources and informed theoretical sampling, category integration and judgements regarding theoretical saturation.

During concurrent grounded‐theory analysis of the first‐round interviews with the initial 23 novice APNs, three additional novice APNs were recruited to develop the emerging categories and examine variation across cases. Their interviews provided further details on the existing categories but did not identify new categories or substantial variations; therefore, no further novice APNs were recruited at this stage. Following preliminary analysis of the longitudinal data, theoretical sampling was extended to experienced APNs to explore later‐stage role development and refine the emerging model. After five retrospective interviews, two additional experienced APNs were recruited. These interviews elaborated the existing categories without requiring modification of the emerging model, after which no additional experienced APNs were recruited.

Final theoretical saturation was assessed through constant comparison across participants, all three longitudinal interview rounds and the retrospective interviews. It was judged to have been reached when the properties and variations of the major categories and the relationships among them were sufficiently developed, and when continued analysis no longer identified new transition stages, critical turning points or relationships among categories requiring modification of the explanatory model. The core process, transition stages, transition outcomes and contextual conditions met these criteria, and no additional substantively distinct transition trajectory was identified. These categories were integrated into an explanatory model that accounted for common and differing patterns across cases and over time.

Data were managed and coded using NVivo 12. All transcripts were analysed in Chinese. Quotations selected for publication were translated into English by the first author and back‐translated into Chinese by the second author. The original and back‐translated versions were compared, and any discrepancies were discussed and revised to ensure semantic equivalence and accuracy, with the research team consulted when necessary.

### 2.5. Reflexivity

Reflexivity was integrated throughout the study. The first author, who conducted all interviews and led the initial analysis, was a PhD candidate in nursing with prior research experience in advanced practice nursing and qualitative methods. This background provided familiarity with APN‐related concepts, role‐development issues and qualitative interviewing, which facilitated rapport with participants and supported in‐depth probing. At the same time, her prior knowledge and research interests could have shaped the questions asked, the meanings attributed to participants’ accounts and the interpretation of emerging categories. The first author maintained reflexive memos throughout data collection and analysis to document prior assumptions, emotional responses, methodological decisions and emerging interpretations. She occupied a position between insider and outsider: she had academic training in nursing and familiarity with APN development in China, but she was not an APN and had no colleague or managerial relationship with participants in the participating institutions.

The second author, a nursing scholar with a PhD in nursing from an overseas university and clinical experience, participated in the coding process and reviewed the evolving coding structure, category boundaries and emerging theoretical interpretations. Her background provided a complementary clinical and international academic perspective, supporting the examination of alternative explanations and the refinement of category properties and relationships. Two other team members, a doctoral candidate in management and a hospital nursing director, contributed disciplinary, managerial and organisational perspectives during regular team discussions. These discussions helped address differences in interpretation, refine major categories and clarify the relationships among categories as the theoretical model developed.

### 2.6. Rigour and Trustworthiness

Charmaz’s criteria of credibility, originality, resonance and usefulness were used to evaluate the quality of the study [25]. Credibility was supported by the longitudinal design, iterative data collection and analysis and constant comparison across participants, interview rounds and data sources. The use of verbatim transcripts, field notes, analytic memos and reflexive memos helped maintain a clear link between participants’ accounts, category development and the emerging theoretical model. Originality was reflected in the development of an explanatory theoretical model that integrated transition stages, trajectories, outcomes, contextual conditions and the recursive process underlying APN role transition. Resonance was supported by using representative quotations, presenting both shared and divergent experiences of role transition and incorporating participant feedback on the findings and interpretations. Usefulness was considered in relation to the model’s potential to inform future research directions, APN professional development and evidence‐based role‐transition support strategies in mainland China.

### 2.7. Ethical Considerations

The study was approved by the Ethics Committee of West China Hospital, Sichuan University (20231992). Prior to the interviews, the participants were informed about the study’s purpose and procedure, as well as the voluntary nature of their participation. Written informed consent was obtained from each participant. To ensure confidentiality, the data were assigned unique codes (ranging from N1 to N33) and access to the data was restricted to the research team only.

## 3. Results

A total of 33 APNs participated in the study. Seven experienced APNs completed one retrospective interview each, while 26 novice APNs who had practised for no more than 1 month at baseline completed three longitudinal interviews over 1 year, yielding 85 interviews in total. Interviews lasted an average of 35 min (range: 31–103 min). Participants were recruited from large public hospitals in five provincial‐level regions of mainland China, although most were practising in Sichuan Province. They represented a range of clinical specialities. Most were female (*n* = 30) and married (*n* = 32). The mean age was 39.6 years (range: 28–50 years), and 10 participants held a master’s degree. Participant characteristics are presented in Table 2.

**TABLE 2:** Characteristics of participants.

Id	Age (years)	Gender	Marital status	Educational level	Nursing experience (years)	Clinical speciality	APN practice experience at first interview	Location
1	32	Female	Married	Bachelor’s degree	14	Gastroenterology	1 month	Sichuan
2	39	Female	Married	Bachelor’s degree	18	Urology	1 month	Sichuan
3	42	Female	Married	Master’s degree	23	Endocrinology	1 month	Sichuan
4	40	Female	Married	Bachelor’s degree	23	Geriatrics	1 month	Sichuan
5	39	Female	Married	Bachelor’s degree	20	General surgery	1 month	Sichuan
6	45	Female	Married	Bachelor’s degree	26	Geriatrics	1 month	Sichuan
7	41	Female	Married	Bachelor’s degree	22	Biological therapy	1 month	Sichuan
8	45	Female	Married	Bachelor’s degree	24	Orthopaedics	1 month	Sichuan
9	37	Female	Married	Master’s degree	15	Endocrinology	1 month	Sichuan
10	50	Female	Married	Bachelor’s degree	26	Gastroenterology	1 month	Sichuan
11	40	Female	Married	Bachelor’s degree	20	Geriatrics	1 month	Sichuan
12	43	Female	Married	Master’s degree	23	Nephrology	1 month	Sichuan
13	45	Female	Married	Bachelor’s degree	23	Thoracic oncology	1 month	Sichuan
14	35	Female	Married	Master’s degree	12	General surgery	1 month	Sichuan
15	39	Female	Married	Bachelor’s degree	27	Cardiovascular surgery	1 month	Sichuan
16	39	Female	Married	Bachelor’s degree	19	General surgery	1 month	Sichuan
17	32	Male	Married	Bachelor’s degree	9	Gastroenterology	1 month	Sichuan
18	37	Male	Married	Bachelor’s degree	18	Rehabilitation medicine	1 month	Sichuan
19	40	Female	Married	Bachelor’s degree	20	Lung cancer	1 month	Sichuan
20	41	Female	Married	Bachelor’s degree	17	Rehabilitation medicine	1 month	Sichuan
21	40	Female	Married	Master’s degree	21	General surgery	1 month	Sichuan
22	41	Female	Married	Bachelor’s degree	16	Thoracic surgery	1 month	Sichuan
23	43	Female	Married	Bachelor’s degree	22	Haemodialysis	1 month	Sichuan
24	44	Female	Married	Master’s degree	22	Cardiology	1 month	Sichuan
25	50	Female	Married	Bachelor’s degree	30	Biliary surgery	1 month	Sichuan
26	43	Female	Married	Bachelor’s degree	25	Cardiology	1 month	Sichuan
27	37	Male	Married	Bachelor’s degree	16	Pain management	5 years	Zhejiang
28	44	Female	Married	Master’s degree	18	Pain management	6 years	Zhejiang
29	35	Female	Married	Master’s degree	9	Neurology	1 year	Chongqing
30	36	Female	Married	Bachelor’s degree	14	Urology	1 year	Chongqing
31	28	Female	Single	Master’s degree	3	ICU	1 year	Shanxi
32	32	Female	Married	Bachelor’s degree	7	Medical oncology	1 year	Shanxi
33	34	Female	Married	Master’s degree	10	ICU	1 year	Hunan

Abbreviation: APN, advanced practice nurse.

### 3.1. Theoretical Model: “The Common yet Different Journey”

**FIGURE 1:**
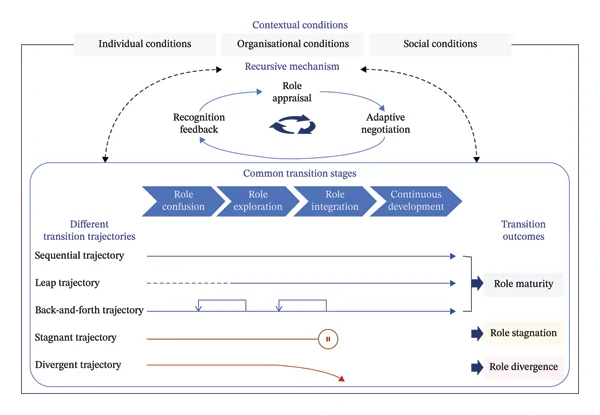
The theoretical model for APN role transition.

Data analysis generated the core category, “the common yet different journey”, which integrated four interrelated components into an explanatory theoretical model of APN role transition: transition stages, transition trajectories, transition outcomes and contextual conditions (Figure 1). Across the data, four commonly observed transition stages were identified: role confusion, role exploration, role integration and continuous development. APNs did not move through these stages uniformly or linearly. Five transition trajectories were identified: sequential, leap, back‐and‐forth, stagnant and divergent, and these trajectories were associated with role maturity, role stagnation, or role divergence. In this model, the “common” aspect refers to the commonly observed transition stages, whereas the “different” aspect refers to variations in how APNs moved through these stages and reached different outcomes. Individual, organisational and social conditions shaped these variations by influencing the recursive transition process.

Throughout the transition process, APNs repeatedly appraised the new role, adjusted their actions and professional interactions and interpreted feedback from relevant stakeholders. Role appraisal concerned the requirements and feasibility of the APN role within the practice context; adaptive negotiation concerned the behavioural, strategic and relational adjustments made to enact the role in practice and recognition feedback concerned responses that affirmed, constrained or redirected role development. Such feedback subsequently informed further role appraisal and adaptive negotiation, sustaining the recursive process.

### 3.2. Transition Stages

Most APNs experienced four commonly observed stages: role confusion, role exploration, role integration and continuous development. Movement among these stages was not always linear.

#### 3.2.1. Role Confusion Stage

On entering the new role, APNs felt as though they had been thrust into an unfamiliar world, experiencing confusion and uncertainty about new areas of practice, responsibilities, tasks and professional relationships.

##### 3.2.1.1. Ambiguous Self‐Positioning

APNs were confused about their professional positioning and occupational identity, with unclear boundaries. This uncertainty led to a temporary loss of self‐identity.

*“When I first moved into this new role, I was not very clear about how to position myself or whether I was expected to manage everything in clinical practice. Sometimes, I would forget and still interact with patients as a ward nurse about issues unrelated to my specialist area.” (N28)*

##### 3.2.1.2. Unclear Job Responsibilities

Most APNs experienced confusion regarding their job content and did not know what to do. The lack of clearly defined responsibilities and tasks was the most prominent characteristic of this stage.

*“At the beginning, there was a sense of confusion and uncertainty about what to do. I was like ‘a headless fly’, aimlessly bumping around. This process was quite painful.” (T1 N6)*

##### 3.2.1.3. Obvious Emotional Fluctuations

In the initial phase, the difficulty in adjusting to the new role led to complex emotions. Participants’ emotions ranged from ambition and enthusiasm to frustration and anxiety.

*“At the beginning, I looked forward to the opportunities that the new role offered. But later I found that there were many difficulties in practice. Now, I often lose sleep, feel anxious.” (T1 N9)*

##### 3.2.1.4. Strong Professional Commitment

The confusion regarding the new role did not diminish the professional commitment of APNs. Choosing to become an APN was a carefully considered decision, and they were determined to invest effort in it.

*“Although I am feeling a bit overwhelmed now, I will resolutely continue on the path I have chosen, with the goal of achieving progress in my professional development.” (T1 N12)*

The shift towards role exploration occurred as APNs appraised the new role and began testing possible ways of enacting it.

#### 3.2.2. Role Exploration Stage

During role exploration, APNs actively sought to understand the new role while navigating competency gaps, competing role demands, blurred work boundaries and uncertainty about their work priorities and professional direction.

##### 3.2.2.1. Competency–Role Mismatch

Although they had extensive clinical experience, they reported that after transitioning into advanced practice roles, their skills in clinical decision‐making, interdisciplinary nursing, nursing research and other areas were insufficient to meet the demands of the role.

*“I don’t work in traditional nursing jobs now. But my previous direct care clinical experience doesn’t match my current position, which requires more critical care management decisions and interdisciplinary nursing.” (T2 N16)*

##### 3.2.2.2. Multiple Role Conflicts

Most APNs struggled to maintain a balance between work, family and academic roles. These inter‐role conflicts sometimes reduced APNs’ engagement in particular roles.

*“Owing to my commitment to exploring a new work model, I am dedicating less time to caring for my family. My child is young and just starts elementary school, while my husband frequently travels for business.” (T2 N20)*

##### 3.2.2.3. Blurred Work Boundaries

At this stage, APNs attempted to explore a variety of tasks; however, due to the lack of clear role boundaries, they were unsure whether they were encroaching on the authority of others. Overlapping responsibilities may result in interpersonal conflicts.

*“There are also nursing group leaders, and some of their responsibilities overlap with ours. What I can do, they can also do.” (T2 N6)*

##### 3.2.2.4. Exploration of Work Methods and Objectives

Due to the complexity and diversity of the role, APNs needed to take time to clarify their current priority tasks and work focus and further establish work objectives and professional directions.

*“Since I am still in the trial phase, there are many things I want to do. I now need to clarify where my focus lies, communicate with physicians and nurses, address patient issues, and establish some process guidelines*…*” (T2 N8)*

For some APNs, practical experience and stakeholder feedback enabled them to reassess the role, clarify their priorities and consolidate a workable approach to practice, thereby supporting movement towards role integration.

#### 3.2.3. Role Integration Stage

During role integration, APNs demonstrated greater stability in how they enacted the role, accompanied by stronger collaborative relationships, role engagement and professional confidence.

##### 3.2.3.1. Clarifying the Practice Model

Through continuous reflection and adjustment, APNs gradually optimised and refined their work methods, developing a practice model that aligned with their personal and professional characteristics while meeting clinical demands, thereby ensuring the efficiency and professionalism of their work.

*“I view this as a period of running-in. After exploring various approaches, I have integrated my previous experiences to develop a process that works for me. I’m clear on what I need to do now, what comes next, the goals I want to achieve in the near future, and so on.” (T2 N7)*

##### 3.2.3.2. Building Cooperative Relationships

APNs proactively communicated with members of the multidisciplinary team, facilitating information sharing and gradually establishing collaborative relationships, such as multidisciplinary consultations and clinics.

*“I participated in a multidisciplinary clinic, where physicians from our department, dietitians from the nutrition department, and I collaboratively managed obesity treatment for patients.” (T2 N3)*

##### 3.2.3.3. High Role Engagement

Many APNs gradually developed a strong sense of role identity and self‐efficacy, leading them to invest more time and energy.

*“I am now fully engaged in this role, and even after work, I still respond to other nurses′ questions.” (T2 N23)*

##### 3.2.3.4. Strong Professional Confidence

The participants reported that they were confident in their professional abilities and clinical decision‐making, handling complex clinical situations with ease and earning recognition from both colleagues and patients.

*“The ability to recognise and think critically is very important. I have made great progress in this area and can now analyse and make decisions quickly.” (T2 N5)*

Increasingly positive recognition reinforced APNs’ perception that the role was workable and prompted further refinement of their practice, thereby supporting movement towards continuous development.

#### 3.2.4. Continuous Development Stage

During continuous development, APNs consolidated their professional strengths, maintained their professional competitiveness and pursued continued personal and professional growth.

##### 3.2.4.1. Developing Professional Strengths

The participants emphasised that, at this stage, they continually accumulated experience and deepened their understanding. They began to identify breakthrough points within their practice areas, developed their own unique advanced nursing philosophy and methods, and formulated relevant research directions.

*“I have been working in the management of chronic kidney disease. Later, I identified several clinical challenges. In response, I proposed the use of bedside ultrasound for early screening of perirenal hematomas to reduce patient safety risks. I then integrated research into this clinical field.” (T3 N12)*

##### 3.2.4.2. Continuous Proactive Learning

They actively participated in a variety of professional training, academic conferences and continuing education programs, keeping themselves informed about the latest developments and research frontiers in their respective fields. They continuously absorbed new knowledge, techniques and methods.

*“The saying goes, ‘You are never too old to learn.’ I am constantly enriching myself. I often review relevant materials after work to accumulate more knowledge, especially interdisciplinary knowledge.” (T3 N8)*

##### 3.2.4.3. Recognition of Role Value

During this phase, they received positive feedback and achieved several work objectives, which allowed them to deeply recognise their value and contributions.

*“A patient presented me with a banner, which is uncommon, as banners were traditionally given only to doctors. The patient′s recognition is the highest form of affirmation for my work.” (T3 N3)*

##### 3.2.4.4. Expanding Role Influence

Some APNs expanded their professional influence within nursing and gained broader recognition across the healthcare sector through activities such as delivering lectures, speaking at conferences, participating in competitions and publishing research articles.

*“I am currently focused on expanding my influence beyond the hospital. I have delivered lectures at other hospitals, including some outside Sichuan Province. Additionally, I have engaged in multicenter research collaborations with these institutions.” (T3 N12)*

Continuous development did not mark the end of role transition; new responsibilities, practice opportunities and professional challenges continued to prompt further role appraisal and adjustment.

### 3.3. Transition Trajectories

Although most APNs encountered the four transition stages, their movement across these stages varied in pace, sequence, direction and continuity. Five transition trajectories were identified: sequential, leap, back‐and‐forth, stagnant and divergent. These trajectories reflected different patterns of movement across transition stages rather than isolated experiences reported at individual time points, showing how the recursive transition process was reinforced, accelerated, interrupted and resumed, persistently constrained, or redirected under differing contextual conditions.

#### 3.3.1. Sequential Transition Trajectory

Eighteen APNs progressed through role confusion, role exploration, role integration and continuous development in sequence, without regression to an earlier stage. This was the most common trajectory, and their role maturity gradually increased over time.

Initially, they described themselves as being in a state of *“confusion and uncertainty”* (*T1 N10, T1 N23)*, and the role transition progressed slowly. However, during the second interview, a turning point was reported, with improvements in *“professional skills and work direction”* (*T2 N8, T2 N20)*, which accelerated the transition. By the final interview, they stated that *“the role had reached a relatively ideal state” (T3 N13).*

In this trajectory, each cycle of role appraisal and adjustment was reinforced by accumulating positive recognition, enabling APNs to build progressively on earlier gains and move steadily through the stages.

#### 3.3.2. Leap Transition Trajectory

Four APNs followed the leap trajectory, characterised by rapid progression through the early stages of transition and early movement towards role integration.

Illustrative accounts from these participants demonstrated how this trajectory unfolded over time. One participant stated, *“Before starting my work, I had already decided what I wanted to do, and I was full of ambition” (T1 N5)*. By the second interview, another participant reported, *“I feel that my current work style has become quite mature, and I have also gained recognition from others” (T2 N7)*. By the final interview, participants had extended their professional activities beyond their own institutions: *“I am no longer confined to the hospital where I work; I frequently visit other hospitals to give presentations” (T3 N18).*

In this trajectory, a clear initial appraisal of the role, prior preparation and supportive practice opportunities enabled APNs to establish workable ways of enacting the role at an early stage. Early positive recognition then reinforced these approaches, accelerating their progression through the early transition stages.

#### 3.3.3. Back‐and‐Forth Transition Trajectory

Five APNs experienced repeated forward and backward movement across the transition stages rather than steady progression, resulting in fluctuations in role maturity over time.

During the second interview, some APNs experienced setbacks in their role practice, leading them to reconsider their approaches and explore alternative ways. One participant described repeated attempts to develop an APN‐led clinic: *“The clinic did not develop as we had hoped because very few patients came. We were repeatedly asked to find ways to promote it. We then set up an online clinic, but there were still very few consultations” (T2 N6).*

Despite these setbacks, participants gradually resumed forward movement. By the third interview, participants described greater confidence and pride in their roles *(T3 N16, T3 N19)*, with one participant reporting, *“I have already realised the value of my role, and everything is developing in a positive way” (T3 N19).*

In this trajectory, unsuccessful attempts and discouraging organisational feedback prompted APNs to reassess the feasibility of the role and their professional direction, sometimes leading them to return to earlier stages. Continued experimentation prompted renewed appraisal and adjustment, while subsequent positive recognition restored confidence in the role and enabled forward movement to resume.

#### 3.3.4. Stagnant Transition Trajectory

Four APNs remained in the role exploration stage for an extended period and did not progress to role integration during the study period.

Although they initially engaged actively in role exploration, by the second interview, some participants felt discouraged. One participant stated, *“cold water had been poured on my enthusiasm” (T2 N31).* One participant mentioned, *“The head nurse seemed to think I was infringing on her authority” (T2 N9),* while another expressed, *“I don’t know what else I can do; it feels like I’m stuck in the same place” (T2 N17).* By the third interview, these difficulties had not been resolved. Participants acknowledged, *“I cannot solve these problems on my own” (T3 N9),* and their roles remained stagnant.

Persistent role ambiguity and negative organisational responses prompted repeated reassessment but constrained APNs’ ability to negotiate role boundaries and establish workable practices. With little positive recognition to reinforce their efforts, movement towards role integration remained stalled.

#### 3.3.5. Divergent Transition Trajectory

Two APNs demonstrated a divergent trajectory, characterised by a gradual departure from expected APN role development patterns over the course of transition.

One participant explained, *“I am scheduled as an APN for only part of the week. On the other days, I may work as a nursing group leader, a staff nurse, or an office nurse—essentially in other roles” (T2 N14)*. By the third interview, this pattern remained evident: *“Sometimes, I work more like the head nurse’s secretary, doing some secretarial work” (T3 N14).*

Another participant described how organisational changes affected the continuation of APN practice: *“After being transferred to another department because of organisational changes, it became difficult for me to continue my advanced practice nursing work, and I did not know how to move forward. In my current department, I can only undertake tasks such as organising research-related documents and assisting with activities assigned by the head nurse” (T2 N1).*

In this trajectory, organisational task allocation constrained opportunities for core APN practice and redirected APNs’ adaptive efforts towards other nursing, managerial or administrative work. Limited opportunities to enact and receive recognition for advanced clinical practice progressively diverted role development from the expected APN role pathway.

### 3.4. Transition Outcomes

Transition outcomes reflected the role states reached through different transition trajectories and were classified into two broad categories: successful and unsuccessful transition. Successful transition was characterised by role maturity, whereas unsuccessful transition included role stagnation and role divergence. These outcomes represented the states observed during the study period rather than definitive endpoints.

#### 3.4.1. Successful Transition

Role maturity was regarded as the favourable outcome of the transition process, indicating that APNs had developed a relatively stable and mature way of enacting the role and could effectively fulfil their role responsibilities. Although the pace of transition varied, most participants had reached the continuous development stage by the end of the study period and were therefore considered to have attained role maturity.

*“I feel that my current job allows me to demonstrate my value, which gives me a sense of job satisfaction.” (T3 N07.*

#### 3.4.2. Unsuccessful Transition

Role stagnation: This outcome was characterised by incomplete consolidation of the APN role. By the end of the study period, APNs were still exploring how to enact the role and therefore had not attained role maturity.

*“I am unsure of the direction my work should take; it feels as though I have been exploring without finding a clear path forward.” (T2 N17)*

Role divergence: This outcome was characterised by a mismatch between the formal APN designation and actual role enactment. Although participants retained the APN title, their everyday responsibilities did not reflect the core functions of advanced practice nursing.

*“Although I hold the position of an APN in the department, I now find myself performing more like the head nurse′s secretary.” (T2 N14)*

### 3.5. Contextual Conditions Influencing Role Transition

**FIGURE 2:**
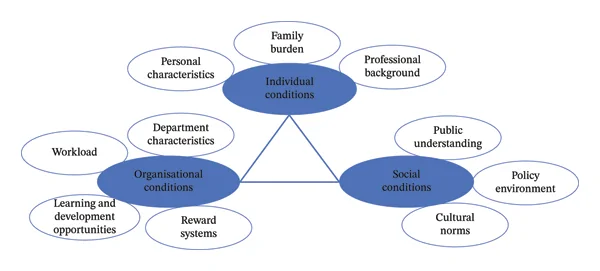
Contextual conditions influencing APN role transition.

APN role transition occurred within individual, organisational and social conditions (Figure 2). By altering the resources, opportunities and legitimacy available to APNs, these conditions facilitated, constrained or redirected the recursive transition process.

#### 3.5.1. Individual Conditions

##### 3.5.1.1. Personal Characteristics

Two participants reported that introversion hindered their communication and collaboration with other healthcare professionals.

*“My biggest obstacle is my introversion, as communicating with doctors requires courage.” (T2 N19)*

Some participants reported that increasing age and reduced physical stamina limited their energy and capacity to cope with demanding workloads.

*“At 43 years old, I find that my physical energy has declined, so I lack the drive.” (T2 N10)*

##### 3.5.1.2. Family Burden

Family burden arising from childcare, eldercare and other family responsibilities required participants to make trade‐offs and constrained the time and energy they could devote to the new role.

*“In the evenings, I would like to reflect on my work or learn new knowledge. However, it is challenging to find the time because I need to help my child with her homework.” (T1 N03)*

##### 3.5.1.3. Professional Background

Educational background appeared to offer different advantages. Participants with postgraduate education described *“a stronger sense of self-identity and professional identity” (T1 N18),* whereas some participants with Bachelor’s degrees highlighted their more extensive clinical experience as *“an advantage in clinical decision-making” (T1 N5).*

Beyond formal education, participants with longer nursing experience regarded their accumulated professional resources as an important foundation for role transition.

*“The professional networks and clinical experience I had accumulated were crucial to my transition into the new role.” (T1 N13)*

Collectively, these individual conditions shaped APNs’ capacity to communicate and collaborate; the time and energy available for role enactment; and the knowledge, experience and professional networks they could draw on during role transition.

#### 3.5.2. Organisational Conditions

##### 3.5.2.1. Department Characteristics

Some participants reported that a high proportion of senior nurses in the department made guidance and coordination more difficult, particularly when established work routines were not easily changed.

*“Sometimes, when I instruct other senior nurses, I can sense the dissatisfaction in their tone of voice. I feel that our relationship has become strained.” (T1 N8)*

Participants also highlighted the influence of the departmental climate.

*“Faced with so many patients with cancer, I sometimes feel powerless, as though I have not been able to help very much. This negative atmosphere affects my work.” (T1 N13)*

##### 3.5.2.2. Workload

High work intensity and frequent overtime added to the strain APNs experienced while adapting to the new role.

*“I work overtime every day. I even wrote the word ‘relax’ and placed it in front of my computer.” (T1 N1)*

##### 3.5.2.3. Learning and Development Opportunities

The organisation provided opportunities to participate in training programmes, academic conferences and research projects, helping APNs update their knowledge, strengthen their skills and broaden their perspectives.

*“The head nurse gave me the opportunity to participate in the hospital’s case competition, which allowed me to demonstrate my clinical skills.” (T2 N12)*

##### 3.5.2.4. Reward Systems

Participants viewed remuneration as an indicator of organisational recognition.

*“I think our remuneration should reflect the nursing department’s recognition of our role. However, my current salary is relatively low.” (T2 N16)*

Together, these organisational conditions shaped the demands, developmental opportunities and recognition surrounding APNs’ role enactment, thereby influencing their ability to negotiate role boundaries and establish workable practices.

#### 3.5.3. Social Conditions

##### 3.5.3.1. Public Understanding

Most participants reported that the public had a limited understanding of the APN role.

*“The public knows very little about the APN role, and patients do not fully trust me.” (T1 N06)*

##### 3.5.3.2. Policy Environment

Participants consistently described insufficient policy and regulatory support as a barrier to APN role development.

*“There is no legal protection for the APN title or prescribing authority, so I feel that I am in an awkward position.” (T1 N18)*

##### 3.5.3.3. Cultural Norms

Participants perceived that traditional stereotypes of nursing constrained the enactment of the APN role. The public commonly associated nurses with routine tasks, such as administering injections and medications, which limited recognition of nurses’ professional expertise and of APNs as clinical experts.

*“Although it is difficult to admit, stereotypical perceptions of nursing are difficult to dispel. The public does not view nursing as a promising profession, nor do they regard me as a clinical expert. I feel somewhat powerless.” (T2 N06)*

Participants also described modesty and humility as influential cultural norms. These norms discouraged some APNs from publicly presenting themselves as advanced practitioners or highlighting their achievements. Although such restraint could help preserve interpersonal harmony, it could also reduce their professional visibility.

*“Our traditional mindset values humility; I would not introduce myself as an APN too openly or in a way that might appear boastful.” (T2 N5)*

Together, these social conditions limited external recognition, formal authority and professional visibility, making it more difficult for the APN role to gain legitimacy and be enacted in practice.

## 4. Discussion

This study developed a substantive theory explaining role transition among hospital‐based APNs in mainland China. Centred on “the common yet different journey”, the theory explains APNs’ common transition stages, different transition trajectories and distinct transition outcomes, and highlights how individual, organisational and social conditions shape APN role transition through a recursive mechanism. Within this emerging APN context, the findings highlight how nurses interpret, enact and negotiate advanced practice roles while role expectations and practice boundaries continue to evolve. The findings may inform targeted transition‐support strategies for APNs in comparable healthcare settings.

The four transition stages identified in this study—role confusion, role exploration, role integration and continuous development—show some conceptual overlap with previous APN transition models. Brown and Olshansky’s model described nurse practitioners’ movement from “limbo” to “legitimacy” [16], while Chang identified the stages of role ambiguity, role acquisition and role implementation [10]. More recently, Hande and Jackson’s grounded theory of nurse practitioner role transition within a fellowship described a five‐phase pathway comprising mapping a path, stepping onto the trailhead, navigating the trailway, gaining traction and summiting [15]. These similarities suggest that APN role transition involves common developmental tasks, including managing uncertainty, clarifying role expectations, developing competence and gaining recognition. However, the meaning and timing of these stages differed in the mainland Chinese context. In more established APN systems, transition may begin before formal role entry, during graduate education, certification or the period between qualification and employment [34]. In this study, transition was more clearly situated after nurses entered institutionally designated advanced practice roles, because APN preparation in mainland China often occurs through structured postregistration training and institutional appointment.

Beyond these overall similarities and differences, several stage‐specific features are noteworthy. Although many participants had substantial prior nursing experience, role confusion arose when they moved into less clearly defined, institutionally designated APN roles, disrupting their established professional positioning. During role exploration, their transition involved not only developing advanced competencies but also constructing role space by negotiating work boundaries, responsibilities and relationships within existing departmental structures [35, 36]. Viewed through role theory, this reflects how role ambiguity, role conflict and unclear boundaries within clinical teams can shape early role transition [37]. Role integration depended on recognition gained through everyday practice, suggesting that role legitimacy was gradually constructed rather than automatically conferred by formal credentials or nationally regulated authority. Continuous development was also not a fixed endpoint, as APNs continued to maintain visibility, demonstrate value and extend influence within an evolving APN system [38]. Our previous phenomenological study described novice APNs’ experiences during the early phase of role transition [31], whereas the present longitudinal analysis examined how role transition developed and varied over time.

The main contribution of this study lies not merely in identifying transition stages, but in revealing their temporal and developmental heterogeneity. While a previous review has noted that APNs may move through transition stages at different rates and in different ways [8], the longitudinal data in this study extend this insight by showing that such variation could be conceptualised as distinct trajectories: sequential, leap, back‐and‐forth, stagnant and divergent. From the perspective of transition theory, this variability indicates that role transition is a dynamic process shaped by facilitating and inhibiting conditions, rather than a linear or unidirectional movement [21]. Similar nonlinear patterns have also been reported in studies of postgraduate nurses and palliative care professionals, where participants moved between phases or diverged towards different outcomes [39, 40]. These diverse trajectories were associated with distinct outcomes, including role maturity, role stagnation and role divergence. Although the divergent trajectory was identified in only two participants and should therefore be interpreted cautiously, it highlights how APN role development may be redirected when the evolving role is not sufficiently sustained or legitimised within the practice context. In this sense, “the common yet different journey” captures both the shared structure and varied pathways of APN role transition.

Beyond the longitudinal trajectory patterns, the recursive mechanism identified in this study provides an explanatory account of how contextual conditions shaped APNs’ movement across stages and trajectories. Through repeated cycles of role appraisal, adaptive negotiation and recognition feedback, APNs interpreted the new role, adjusted their actions and relationships and responded to feedback from relevant stakeholders. When role expectations were clear, practice opportunities were available and feedback was positive, this cycle supported movement towards role integration, continuous development and role maturity. Conversely, unresolved role ambiguity, limited opportunities, weak recognition or persistent role conflict could disrupt the cycle, leading to back‐and‐forth movement, stagnation or divergence. This process particularly resonates with symbolic interactionism, which highlights how role meaning, professional value and legitimacy are formed and revised through social interaction and feedback [41].

Individual conditions shaped APNs’ readiness for role transition and their capacity to sustain role development. Prior clinical experience, communication style, educational background, family responsibilities and physical or emotional resources affected how APNs responded to role demands [42–45]. The influence of education appeared complex. Graduate‐prepared APNs may have advantages in evidence‐based practice and academic development, whereas APNs with extensive clinical experience may demonstrate greater confidence in clinical management and decision‐making [18]. However, this finding should not be interpreted as diminishing the importance of graduate education. Rather, it highlights the need for transition pathways that progressively strengthen both advanced clinical competence and academic preparation in line with international expectations for APN development. Healthcare organisations should consider these individual differences when selecting, appointing and supporting APNs, and provide differentiated support according to APNs’ prior experience, educational preparation and developmental needs [46].

Organisational conditions were also central to APN role transition. Supportive departmental climates, developmental resources, recognition systems and opportunities for meaningful advanced practice facilitated role integration and continuous development [43, 47–49]. Conversely, unclear role boundaries and hierarchical relationships could make role enactment more difficult. In some departments, the presence of many senior nurses appeared to complicate APNs’ role development. This may have reflected dissatisfaction or perceived threats to existing status among some senior team members, particularly when the emerging APN role altered informal authority within the team or overlapped with existing responsibilities. Similar interpersonal tensions have been reported in previous APN transition research, in which participants described being derided or criticised for their talents or achievements, and experiencing resistance from senior nurses [28]. These findings suggest that APN implementation should involve not only individual APNs, but also key stakeholders within clinical teams, so that expectations, responsibilities and collaborative relationships can be clarified collectively [50].

At the broader contextual level, regulatory gaps, limited public understanding and insufficient recognition of advanced nursing practice constrained APN role transition [42, 51–53]. A notable finding was that some APNs were reluctant to actively display their achievements, which may be related to cultural norms valuing modesty. Although this may reflect professional humility, it could also limit the visibility of APN contributions and reduce opportunities for wider recognition [54]. Viewed through a professional socialisation lens, this suggests that APN role transition also depends on recognition of advanced nursing practice within the wider professional and sociocultural environment, highlighting the need for broader professional and public efforts to enhance its visibility and legitimacy [55].

## 5. Implications for Nursing Management

These findings carry several implications for nursing management. First, given the heterogeneous trajectories observed, institutions should establish structured and adaptive transition‐support systems that recognise both the shared developmental needs and trajectory‐specific challenges of APNs. Institutions may consider incorporating early transition assessment and feedback processes to identify APNs who demonstrate stagnant or divergent trajectories and provide timely, tailored support. APNs experiencing stagnant trajectories may benefit from interventions that enhance role clarity, competency development and opportunities for advanced practice, whereas those showing divergent trajectories may require review of task allocation and organisational support to prevent further divergence from the intended APN role pathway. Second, managers should establish mechanisms to document and evaluate APN contributions to patient outcomes, care quality, service improvement and workforce development. Such evidence is critical for demonstrating APN value in mainland China and may provide an empirical basis for future policy development, including education standards, credentialing mechanisms, scope of practice, remuneration and career progression. Professional organisations and healthcare institutions should further improve public and professional understanding of APNs through outcome reporting, exemplary practice cases, interdisciplinary forums and public education campaigns.

## 6. Limitations

This study has several limitations. First, although participants were recruited from five provincial‐level regions of mainland China and varied in clinical speciality, educational background and professional experience, most were practising in Sichuan Province. The findings should therefore not be interpreted as representing all hospital‐based APNs across mainland China. However, healthcare delivery in mainland China is shaped by a common national policy framework, and APN roles are commonly situated in large public hospitals, which often share broadly similar organisational structures and operating mechanisms. These shared system‐level and organisational features may create a degree of contextual similarity across regions and suggest that the model may be relevant to APNs working in comparable hospital settings. Nevertheless, regional differences in APN role development, institutional arrangements and resource allocation may not have been fully captured. The transferability of the model across regions, practice settings and more established APN systems therefore requires further examination.

Second, the sample was predominantly female, which partly reflects the gender composition of the nursing workforce and the limited availability of male APNs. Although the three male participants provided detailed accounts and offered some representation of male perspectives, their small number limited exploration of potentially gender‐specific barriers, expectations and identity negotiations.

Third, most participants were married and middle‐aged. This profile may reflect the career pathway into advanced practice nursing in China, where nurses commonly enter APN roles through in‐service training after accumulating substantial clinical experience. Although four participants were aged 32 years or younger, the experiences of younger, unmarried and early‐career APNs may not have been fully represented.

Fourth, the inclusion of experienced APNs may have introduced selection and recall biases. Although stagnant, divergent and back‐and‐forth trajectories were identified from the longitudinal data of novice APNs, experienced APNs who had remained in advanced practice roles for more than 1 year may have represented those who had adapted relatively successfully. Their accounts may therefore have over‐represented positive transitions and under‐represented challenging or rough experiences. In addition, their retrospective descriptions of early transition may have been influenced by later experiences. To mitigate this limitation, experienced APNs were asked to focus on their first year of practice, and temporal probing was used to support recall of key events and changes over time. Their accounts were used alongside the longitudinal data from novice APNs to elaborate, compare and refine the emerging categories. Furthermore, the divergent trajectory was based on a small number of cases and requires further examination across diverse APN practice contexts.

Future research should include more geographically and demographically diverse samples of hospital‐based APNs from role entry to further examine the transferability of the model and the conditions under which different transition trajectories emerge. Building on the proposed theoretical model, future research could develop transition assessment tools to facilitate early identification of APNs experiencing or at risk of developing stagnant or divergent trajectories and inform targeted interventions. Quantitative and mixed‐methods studies should examine the relationships proposed in the substantive theory and identify factors influencing APN role transition, while intervention and implementation studies are needed to evaluate its practical utility and inform the development of evidence‐based support strategies.

## 7. Conclusions

This study developed a substantive theory explaining role transition among hospital‐based APNs in the mainland Chinese hospital contexts represented in this study. Centred on “the common yet different journey”, the theory offers a contextually grounded explanation of how APNs move into and enact advanced practice roles. The findings may inform the development of staged, tailored and context‐sensitive transition‐support strategies, as well as future APN role and policy development in mainland China.

## Funding

No funding was received for this manuscript.

## Conflicts of Interest

The authors declare no conflicts of interest.

## Supporting Information

Additional supporting information can be found online in the Supporting Information section.

## Supporting information


**Supporting Information** Supporting Information 1: COREQ checklist. Supporting Information 2: The interview guide.

## Data Availability

The data that support the findings of this study are available upon request from the corresponding author. The data are not publicly available due to privacy or ethical restrictions.

## References

[1] World Health Organization , State of the World’s Nursing 2020: Investing in Education, Jobs, and Leadership, 2020, https://apps.who.int/iris/handle/10665/331677.

[2] Fajarini M. , Setiawan A. , Sung C. M. et al., Effects of Advanced Practice Nurses on health-care Costs, Quality of Care, and Patient well-being: a meta-analysis of Randomized Controlled Trials, International Journal of Nursing Studies. (2025) 162, 10.1016/j.ijnurstu.2024.104953. 39586168

[3] Scanlon A. , Murphy M. , Smolowitz J. , and Lewis V. , Low- and Lower middle-income Countries Advanced Practice Nurses: an Integrative Review, International Nursing Review. (2020) 67, no. 1, 19–34, 10.1111/inr.12536. 31364775

[4] International Council of Nurses , Guidelines on Advanced Practice Nursing, 2020, https://www.icn.ch/sites/default/files/2023-04/ICN_APN%20Report_EN.pdf.

[5] Allsop S. , Morphet J. , Lee S. , and Cook O. , Exploring the Roles of Advanced Practice Nurses in the Care of Patients Following Fragility Hip Fracture: a Systematic Review, Journal of Advanced Nursing. (2021) 77, no. 5, 2166–2184, 10.1111/jan.14692. 33320350

[6] McMenamin A. , Turi E. , Schlak A. , and Poghosyan L. , A Systematic Review of Outcomes Related to Nurse Practitioner-Delivered Primary Care for Multiple Chronic Conditions, Medical Care Research and Review. (2023) 80, no. 6, 563–581, 10.1177/10775587231186720. PMC10784406 37438917

[7] Turi E. , McMenamin A. , Kueakomoldej S. , Kurtzman E. , and Poghosyan L. , The Effectiveness of Nurse Practitioner Care for Patients with Mental Health Conditions in Primary Care Settings: a Systematic Review, Nursing Outlook. (2023) 71, no. 4, 10.1016/j.outlook.2023.101995. PMC10592550 37343483

[8] Li Y. , Wang C. , Tan W. , and Jiang Y. , The Transition to Advanced Practice Nursing: a Systematic Review of Qualitative Studies, International Journal of Nursing Studies. (2023) 144, 10.1016/j.ijnurstu.2023.104525. 37263057

[9] Cheng J. F. , Wang T. J. , Huang X. Y. , and Han H. C. , First-Year Experience of Transitioning from Registered Nurse to Nurse Practitioner, J Am Assoc Nurse Pract. (2022) 34, no. 8, 978–990, 10.1097/jxx.0000000000000750. 36330551

[10] Chang W. C. , Mu P. F. , and Tsay S. L. , The Experience of Role Transition in Acute Care Nurse Practitioners in Taiwan Under the Collaborative Practice Model, Journal of Nursing Research. (2006) 14, no. 2, 83–92, 10.1097/01.jnr.0000387566.34318.b2. 16741858

[11] Toniolo J. , Ngoungou E. B. , Ategbo S. et al., Implementation Strategy for Advanced Practice Nursing in Gabon: a Multicenter mixed-method Study, International Nursing Review. (2024) 71, no. 2, 326–334, 10.1111/inr.12903. 37962067

[12] Barnes H. , Exploring the Factors that Influence Nurse Practitioner Role Transition, The Journal for Nurse Practitioners. (2015) 11, no. 2, 178–183, 10.1016/j.nurpra.2014.11.004. PMC4323084 25685113

[13] Ares T. L. , Role Transition After Clinical Nurse Specialist Education, Clinical Nurse Specialist. (2018) 32, no. 2, 71–80, 10.1097/nur.0000000000000357. 29419579

[14] Chen Y. J. and Lin K. P. , Association Among Work Characteristics, Role Transition, and Job Burnout in Nurse Practitioners in Taiwan, Inquiry. (2022) 59, 10.1177/00469580221081403. PMC8921748 35274551

[15] Hande K. and Jackson H. , Navigating the Pathway to Advanced Practice: a Grounded Theory of Nurse Practitioner Role Transition in a Fellowship, Journal of the American Association of Nurse Practitioners. (2024) 36, no. 4, 221–232, 10.1097/JXX.0000000000001000. 38320261

[16] Brown M. A. and Olshansky E. F. , From Limbo to Legitimacy: a Theoretical Model of the Transition to the Primary Care Nurse Practitioner Role, Nursing Research. (1997) 46, no. 1, 46–51, 10.1097/00006199-199701000-00008. 9024424

[17] Thompson A. , An Educational Intervention to Enhance Nurse Practitioner Role Transition in the First Year of Practice, J Am Assoc Nurse Pract. (2019) 31, no. 1, 24–32, 10.1097/jxx.0000000000000095. 30211782

[18] Liu Y. W. , Hei L. X. , Gong R. R. , Ning N. , and Jiang Y. , Reflection on the Way of Selecting and Training of Advanced Practice Nurse Candidate, Chinese Journal of Nursing Education. (2023) 20, no. 9, 1147–1152, 10.3761/j.issn.1672-9234.2023.09.021.

[19] Wong F. K. Y. , Development of Advanced Nursing Practice in China: Act Local and Think Global, International Journal of Nursing Science. (2018) 5, no. 2, 101–104, 10.1016/j.ijnss.2018.03.003. PMC6626258 31406809

[20] Zhan Q. , Shang S. , Li W. , and Chen L. , Bridging the GP Gap: Nurse Practitioners in China, Lancet. (2019) 394, no. 10204, 1125–1127, 10.1016/s0140-6736(19)32209-3. 31571590

[21] Meleis A. I. , Sawyer L. M. , Im E. O. , Hilfinger Messias D. K. , and Schumacher K. , Experiencing Transitions: an Emerging middle-range Theory, ANS. Advances in nursing science. (2000) 23, no. 1, 12–28, 10.1097/00012272-200009000-00006. 10970036

[22] Lin S. , Chen S. , Tu Q. et al., Barriers and Facilitators to the Formation of Professional Identity Among Nursing Students: a four-year Longitudinal Qualitative Study, Nurse Education Today. (2023) 134, 10.1016/j.nedt.2023.106087. 38232627

[23] Wang W. D. , Hu L. H. , Han J. , and Cai C. , Survey of the Public′S Perception on Image of Nurse Role and Nursing Profession, J Nurs Sci. (2023) 38, no. 07, 1–5, 10.3870/j.issn.1001-4152.2023.07.001.

[24] Charmaz K. , Constructing Grounded Theory, 2014, 2nd edition, SAGE Publications, London.

[25] Charmaz K. and Thornberg R. , The Pursuit of Quality in Grounded Theory, Qualitative Research in Psychology. (2021) 18, no. 3, 305–327, 10.1080/14780887.2020.1780357.

[26] Tong A. , Sainsbury P. , and Craig J. , Consolidated Criteria for Reporting Qualitative Research (COREQ): a 32-item Checklist for Interviews and Focus Groups, International Journal for Quality in Health Care. (2007) 19, no. 6, 349–357, 10.1093/intqhc/mzm042. 17872937

[27] Gong R. R. , He L. X. , Chen J. L. , Zhou X. H. , and Jiang Y. , Construction of Core Competence Framework for Advanced Practice Nurse, Chinese Nursing Management. (2023) 23, no. 5, 654–659, 10.3969/j.issn.1672-1756.2023.05.004.

[28] MacLellan L. , Higgins I. , and Levett-Jones T. , An Exploration of the Factors that Influence Nurse Practitioner Transition in Australia: a Story of Turmoil, Tenacity, and Triumph, Journal American Association Nurse Practice. (2017) 29, no. 3, 149–156, 10.1002/2327-6924.12423. 27860414

[29] Johnson A. H. and Harrison T. C. , Advanced Practice Registered Nurse Transition to Practice in the Long-Term Care Setting: an Ethnography, Global Qualitative Nursing Research. (2022) 9, 10.1177/23333936221108701. PMC9272163 35832603

[30] Angelow A. M. , The Lived Experience of Nurse Practitioner Role Transition: A Phenomenology Study, 2018, University of Northern Colorado, Colorado.

[31] Tan W. , Hu Q. , Liu S. , Wang C. , Wang L. , and Jiang Y. , Navigating the Uncharted waters′- Transition Experiences of Novice Advanced Practice Nurses in China: a Descriptive Phenomenological Study, BMC Nursing. (2026) 25, no. 1, 10.1186/s12912-026-04626-8. PMC13234987 41943038

[32] Grossoehme D. and Lipstein E. , Analyzing Longitudinal Qualitative Data: the Application of Trajectory and Recurrent cross-sectional Approaches, BMC Research Notes. (2016) 9, no. 1, 10.1186/s13104-016-1954-1. PMC4776420 26936266

[33] Nevedal A. L. , Ayalon L. , and Briller S. H. , A Qualitative Evidence Synthesis Review of Longitudinal Qualitative Research in Gerontology, The Gerontologist. (2019) 59, no. 6, e791–e801, 10.1093/geront/gny134. 30395232

[34] Sulosaari V. , Blaževičienė A. , Bragadóttir H. et al., A Comparative Review of Advanced Practice Nurse Programmes in the Nordic and Baltic Countries, Nurse Education Today. (2023) 127, 10.1016/j.nedt.2023.105847. 37216703

[35] Duke C. R. , The Lived Experience of Nurse Practitioner Graduates′ Transition to Hospital-Based Practice, 2010, East Carolina University, Carolina.

[36] Chau J. P. C. , Lo S. H. S. , Lam S. K. Y. , Saran R. , and Thompson D. R. , Critical Elements in Nursing Graduates′ Transition to Advanced Practice Roles and Their Perceived Impact on Patient Care: an Exploratory, Descriptive Study of Graduates′ and Their Managers′ Perceptions, BMC Nursing. (2022) 21, no. 1, 10.1186/s12912-022-00907-0. PMC9121560 35590330

[37] Kahn R. L. , Wolfe D. M. , Quinn R. P. , Snoek J. D. , and Rosenthal R. A. , Organizational Stress: Studies in Role Conflict and Ambiguity, 1964.

[38] Graham D. , Novice Nurse Practitioners’ Perceptions of Role Transition, 2020, Western Connecticut State University, Connecticut.

[39] Mota Vargas R. , Mahtani-Chugani V. , Solano Pallero M. , Rivero Jiménez B. , Cabo Domínguez R. , and Robles Alonso V. , The Transformation Process for Palliative Care Professionals: the Metamorphosis, a Qualitative Research Study, Palliative Medicine. (2016) 30, no. 2, 161–170, 10.1177/0269216315583434. 25895537

[40] Yao H. , Xie C. , Luo M. , and Luo Y. , A level-breaking Adventure Game’: A Grounded Theory Study of Postgraduate Nurses′ Academic Growth Trajectory, Nurse Education Today. (2025) 146, 10.1016/j.nedt.2024.106520. 39616708

[41] Blumer H. , Symbolic Interactionism: Perspective and Method, 1986, Univ of California Press.

[42] Torrens C. , Campbell P. , Hoskins G. et al., Barriers and Facilitators to the Implementation of the Advanced Nurse Practitioner Role in Primary Care Settings: a Scoping Review, International Journal of Nursing Studies. (2020) 104, 10.1016/j.ijnurstu.2019.103443. 32120089

[43] El Hussein M. T. and Ha C. , Facilitators and Barriers to the Transition from Registered Nurse to Nurse Practitioner in Canada, J Am Assoc Nurse Pract. (2023) 35, no. 6, 359–365, 10.1097/jxx.0000000000000868. 37141458

[44] Klein C. J. , Weinzimmer L. G. , Cooling M. , Lizer S. , Pierce L. , and Dalstrom M. , Exploring Burnout and Job Stressors Among Advanced Practice Providers, Nursing Outlook. (2020) 68, no. 2, 145–154, 10.1016/j.outlook.2019.09.005. 31708107

[45] Rakhab A. , Jackson C. , Nilmanat K. , Butterworth T. , and Kane R. , Factors Supporting Career Pathway Development Amongst Advanced Practice Nurses in Thailand: a cross-sectional Survey, International Journal of Nursing Studies. (2021) 117, 10.1016/j.ijnurstu.2021.103882. 33621719

[46] Djukic M. and Fletcher J. , Factors Associated with New Nurses’ Career Choice as Advanced Practice Nurses: Implications for Managing Organizational Turnover, Applied Nursing Research. (2022) 63, 10.1016/j.apnr.2021.151541. 35034710

[47] Faraz A. , Facilitators and Barriers to the Novice Nurse Practitioner Workforce Transition in Primary Care, J Am Assoc Nurse Pract. (2019) 31, no. 6, 364–370, 10.1097/jxx.0000000000000158. 30681654

[48] Kim Y. H. , Shin S. I. , Kim H. K. , Jun M. , and Wreen M. , Advanced Practice Nurses′ Organisation Commitment: Impact of Job Environment, Job Satisfaction, and Person-Organisation Fit, Asian Nursing Research. (2023) 17, no. 2, 91–101, 10.1016/j.anr.2023.03.002. 36997063

[49] Wright M. M. M. , Kvist T. A. , Imeläinen S. M. , and Jokiniemi K. S. , Continuing Education for Advanced Practice Nurses: a Scoping Review, Journal of Advanced Nursing. (2024) 80, no. 8, 3037–3058, 10.1111/jan.15911. 37902130

[50] Wood E. , King R. , Robertson S. , Senek M. , Tod A. , and Ryan T. , Sources of Satisfaction, Dissatisfaction and well-being for UK Advanced Practice Nurses: a Qualitative Study, Journal of Nursing Management. (2021) 29, no. 5, 1073–1080, 10.1111/jonm.13245. 33404130

[51] Dai M. , Hu L. , Sun L. , Zhong Y. , and Li C. , A cross-sectional Study on Chinese Senior Nurses′ Knowledge and Attitudes Toward Nurse Practitioners, BMC Nursing. (2024) 23, no. 1, 10.1186/s12912-024-02257-5. PMC11346258 39183298

[52] Suzuki M. , Harada N. , Honda K. et al., Facilitators and Barriers in Implementing the Nurse Practitioner Role in Japan: a cross-sectional Descriptive Study, International Nursing Review. (2024) 71, no. 2, 291–298, 10.1111/inr.12790. 35839821

[53] Zhou L. , Godsey J. A. , Kallmeyer R. , Hayes T. , and Cai E. , Public Perceptions of the Brand Image of Nursing: Cross-Cultural Differences Between the United States and China, Nursing Outlook. (2024) 72, no. 5, 10.1016/j.outlook.2024.102220. 38878616

[54] Xiong M. , Wang F. , and Cai R. , Development and Validation of the Chinese Modesty Scale (CMS), Frontiers in Psychology. (2018) 9, 10.3389/fpsyg.2018.02014. PMC6206587 30405496

[55] Clouder L. , Becoming Professional: Exploring the Complexities of Professional Socialization in Health and Social Care, Learning in Heatlh and Social Care. (2003) 2, no. 4, 213–222, 10.1046/j.1473-6861.2003.00052.x.

