# Supplementary material for: The Common yet Different Journey: Role Transition Among Hospital‐Based Advanced Practice Nurses in Mainland China: A Grounded Theory Study
Source: J Nurs Manag. 2026 Sep 25;2026:1897994. doi: 10.1155/jonm/1897994 (PMC13613938; doi:10.1155/jonm/1897994)
Supplement: Supplementary file 1 [file JONM-2026-1897994-s001.docx]

**The common yet different journey: Transition experiences of advanced practice nurses in China—a grounded theory study**

**Supporting Information 1: COREQ checklist**

| **No.** | **Item** | **Guide questions/description** | **Page No.** |
| --- | --- | --- | --- |
| Domain 1: Research team and reflexivity | | |  |
| Personal Characteristics | | |  |
| 1 | Interviewer/facilitator | Which author/s conducted the interview or focus group? | 5 |
| 2 | Credentials | What were the researcher's credentials? e.g. *PhD, MD* | 6-7 |
| 3 | Occupation | What was their occupation at the time of the study? | 6-7 |
| 4 | Gender | Was the researcher male or female? | 6-7 |
| 5 | Experience and training | What experience or training did the researcher have? | 6-7 |
| Relationship with participants | | |  |
| 6 | Relationship established | Was a relationship established prior to study commencement? | 6 |
| 7 | Participant knowledge of the interviewer | What did the participants know about the researcher? e.g. *personal goals, reasons for doing the researc*h | 5 |
| 8 | Interviewer characteristics | What characteristics were reported about the interviewer/facilitator? e.g. *Bias, assumptions, reasons and interests in the research topic* | 6-7 |
| Domain 2: study design | | |  |
| Theoretical framework | | |  |
| 9 | Methodological orientation and Theory | What methodological orientation was stated to underpin the study? *e.g. grounded theory, discourse analysis, ethnography, phenomenology, content analysis* | 4 |
| Participant selection | | |  |
| 10 | Sampling | How were participants selected? *e.g. purposive, convenience, consecutive, snowball* | 5 |
| 11 | Method of approach | How were participants approached? e*.g. face-to-face, telephone, mail, email* | 5 |
| 12 | Sample size | How many participants were in the study? | 7 |
| 13 | Non-participation | How many people refused to participate or dropped out? Reasons? | 7 |
| Setting | | |  |
| 14 | Setting of data collection | Where was the data collected? e*.g. home, clinic, workplace* | 5 |
| 15 | Presence of non-participants | Was anyone else present besides the participants and researchers? | 5 |
| 16 | Description of sample | What are the important characteristics of the sample? *e.g. demographic data* | 8 |
| Data collection | | |  |
| 17 | Interview guide | Were questions, prompts, guides provided by the authors? Was it pilot tested? | 5, Supporting Information 2 |
| 18 | Repeat interviews | Were repeat interviews carried out? If yes, how many? | 7 |
| 19 | Audio/visual recording | Did the research use audio or visual recording to collect the data? | 5 |
| 20 | Field notes | Were field notes made during and/or after the interview or focus group? | 5 |
| 21 | Duration | What was the duration of the interviews or focus group? | 8 |
| 22 | Data saturation | Was data saturation discussed? | 6 |
| 23 | Transcripts returned | Were transcripts returned to participants for comment and/or correction? | 7 |
| **Domain 3: analysis and findings** | | |  |
| Data analysis | | |  |
| 24 | Number of data coders | How many data coders coded the data? | 7 |
| 25 | Description of the coding tree | Did authors provide a description of the coding tree? | 9 |
| 26 | Derivation of themes | Were themes identified in advance or derived from the data? | 6 |
| 27 | Software | What software, if applicable, was used to manage the data? | 6 |
| 28 | Participant checking | Did participants provide feedback on the findings? | 7 |
| Reporting | | |  |
| 29 | Quotations presented | Were participant quotations presented to illustrate the themes / findings? Was each quotation identified? e*.g. participant number* | 10-19 |
| 30 | Data and findings consistent | Was there consistency between the data presented and the findings? | 10-19 |
| 31 | Clarity of major themes | Were major themes clearly presented in the findings? | 10-19 |
| 32 | Clarity of minor themes | Is there a description of diverse cases or discussion of minor themes? | 10-19 |

**Supporting Information 2: The interview guide**

**T1 interview guide**

| **Interview stage** | **Primary questions** |
| --- | --- |
| Opening question | - Could you share your experience of first entering the Advanced Practice Nurse role? |
| Continued question | - When you first transitioned into the APN role, what expectations and feelings did you have? |
|  | - What changes have occurred in your work responsibilities, work patterns, professional relationships, or role identity, and how have these changes affected you? |
|  | - How would you describe your current work situation as an APN? |
|  | - What challenges have you faced so far, and how have you responded to them? |
|  | - What support have you received, and what additional support do you need? |
|  | - Could you describe areas of professional growth and competencies that require further development? |
| Closing question | - Is there anything else you would like to share about your transition experience? |

**T2 interview guide**

| **Interview stage** | **Primary questions** |
| --- | --- |
| Opening question | - Now that you have practised as an APN for approximately 6 months, how would you describe your transition experience so far? |
| Continued question | - Since the previous interview, what changes have occurred in your APN practice, and how have these changes affected you? |
|  | - How has your work situation changed, particularly in terms of responsibilities, work patterns, and professional relationships? |
|  | - Have you experienced any critical events or turning points during this period? |
|  | - What challenges have you faced during this period, and how have you responded to them? |
|  | - What support have you received during this period, and what additional support do you need? |
|  | - Could you describe any professional growth you have experienced and any competencies that still require further development? |
| Closing question | - Is there anything else you would like to share about your transition experience? |

**T3 interview guide**

| **Interview stage** | **Primary questions** |
| --- | --- |
| Opening question | - Now that you have practised as an APN for approximately 12 months, how would you describe your overall transition experience? |
| Continued question | - Since the previous interview, what changes have occurred in your APN practice, and how have these changes affected you? |
|  | - How would you describe your current work situation, particularly in terms of responsibilities, work patterns, and professional relationships? |
|  | - Looking back over the past year, were there any critical events or turning points in your transition? |
|  | - What challenges have you faced during this period, and how have you responded to them? |
|  | - What support have you received, and what additional support would have helped your transition? |
|  | - Could you describe your professional growth over the past year and identify competencies that still require further development? |
|  | - What are your professional development goals as an APN moving forward? |
| Closing question | - Is there anything else you would like to share about your transition experience? |

**Retrospective interview guide**

| **Interview stage** | **Primary questions** |
| --- | --- |
| Opening question | - Could you share your experience of transitioning into the Advanced Practice Nurse role, especially during your first year of practice? |
| Continued question | - When you first entered the APN role, what were your expectations and initial feelings? |
|  | - What changes occurred in your work responsibilities, work patterns, professional relationships, or role identity during the transition? - How did these changes affect you personally and professionally? |
|  | - Can you recall any critical events or turning points during your first year as an APN? |
|  | - What challenges did you face during the transition? - How did you respond to or overcome these challenges? |
|  | - What support did you receive from colleagues, managers, organisations, or others? - What additional support would have helped you during the transition? |
|  | - How would you describe your current work situation as an APN, including your responsibilities and practice patterns? - Looking back, what areas of professional growth have you experienced, and what competencies still require further development? |
|  | - What are your professional development goals as an APN moving forward? |
| Closing question | - Is there anything else you would like to share about your transition experience? |
